# In Vivo ^1^H MR Spectroscopy of Biliary Components of Human Gallbladder at 7T

**DOI:** 10.1002/jmri.27207

**Published:** 2020-06-05

**Authors:** Martin Gajdošík, Marek Chmelík, Emina Halilbasic, Lorenz Pfleger, Radka Klepochová, Michael Trauner, Siegfried Trattnig, Martin Krššák

**Affiliations:** ^1^ High‐field MR Centre, Department of Biomedical Imaging and Image‐guided Therapy Medical University of Vienna Vienna Austria; ^2^ Division of Endocrinology and Metabolism, Department of Internal Medicine III Medical University of Vienna Vienna Austria; ^3^ Department of Biomedical Engineering Columbia University Fu Foundation School of Engineering and Applied Science New York New York USA; ^4^ Faculty of Healthcare University of Prešov Prešov Slovakia; ^5^ Department of Radiology General Hospital of Levoča Levoča Slovakia; ^6^ Division of Gastroenterology and Hepatology, Department of Internal Medicine III Medical University of Vienna Vienna Austria; ^7^ Medical University of Vienna, Christian Doppler Laboratory for Clinical Molecular Imaging MOLIMA Vienna Austria

**Keywords:** in vivo MRS, gallbladder, bile, T2 relaxation, 7T, single‐voxel spectroscopy

## Abstract

**Background:**

Previous *in vivo* proton MR spectroscopy (MRS) studies have demonstrated the possibility of quantifying amide groups of conjugated bile acids (NHCBA), olefinic lipids and cholesterol (OLC), choline‐containing phospholipids (CCPLs), taurine and glycine conjugated bile acids (TCBA, GCBA), methylene group of lipids (ML), and methyl groups of bile acids, lipids, and cholesterol (BALC1.0, BALC0.9, and TBAC) in the gallbladder, which may be useful for the study of cholestatic diseases and cholangiopathies. However, these studies were performed at 1.5T and 3T, and higher magnetic fields may offer improved spectral resolution and signal intensity.

**Purpose:**

To develop a method for gallbladder MRS at 7T.

**Study Type:**

Retrospective, technical development.

**Population:**

Ten healthy subjects (five males and five females), two patients with primary biliary cholangitis (PBC) (one male and one female), and one patient with primary sclerosing cholangitis (PSC) (female).

**Field Strength/Sequence:**

Free‐breathing single‐voxel MRS with a modified stimulated echo acquisition mode (STEAM) sequence at 7T.

**Assessment:**

Postprocessing was based on the T_2_ relaxation of water in the gallbladder and in the liver. Concentrations of biliary components were calculated using water signal. All data were corrected for T_2_ relaxation times measured in healthy subjects.

**Statistical Tests:**

The range of T_2_ relaxation time and concentration per bile component, and the resulting mean and standard deviation, were calculated.

**Results:**

The concentrations of gallbladder components in healthy subjects were: NHCBA: 93 ± 66 mM, OLC: 154 ± 124 mM, CCPL: 42 ± 17 mM, TCBA: 48 ± 35 mM, GCBA: 67 ± 32 mM, ML: 740 ± 391 mM, BALC1.0: 175 ± 92 mM, BALC0.9: 260 ± 138 mM, and TBAC: 153 ± 90 mM. Mean concentrations of all bile components were found to be lower in patients.

**Data Conclusion:**

This work provides a protocol for designing future MRS investigations of the bile system *in vivo*.

**Evidence Level:**

2

**Technical Efficacy Stage:**

1

BILE IS A COMPLEX SECRETION that originates from hepatocytes in the liver and is modified by the bile duct epithelium. It consists of water in which are dissolved a number of endogenous solid constituents including bile salts, phospholipids, cholesterol, bilirubin, amino acids, and steroids.^1^ Bile is concentrated in the gallbladder from which it reaches the intestinal lumen.^1^ Bile is the major excretory route for lipophilic substances as well as other endogenous substrates, such as bilirubin and bile salts. Bile salts are the major organic solutes in bile and normally function to emulsify dietary fats and facilitate their absorption. Bile is also the major route for elimination of cholesterol and protects the organism from enteric infections, and some biliary compounds (eg, bile salts, bilirubin) undergo an enterohepatic circulation.^1^

Changes in bile composition are not only relevant for gallstone formation but may also play an important role in the pathogenesis and treatment of chronic inflammatory bile duct diseases.^2^ Importantly, the stimulation of phospholipid secretion may be a key therapeutic mechanism of several established and new drugs for treatment of cholangiopathies such as primary biliary cholangitis (PBC) and primary sclerosing cholangitis (PSC).^2^

Bile can be characterized *in vitro* in the micellar phase with proton nuclear magnetic resonance spectroscopy (MRS) analysis.^3^, ^4^, ^5^, ^6^ Pioneer work in gallbladder *in vivo* MRS in humans was published by Prescot et al,^7^ where choline‐containing phospholipid signal (CCPL, 3.22 ppm) at a magnetic field strength (B_0_) of 1.5T was detected. Künnecke et al showed reliable detection of several other bile components in monkeys at 4.7T,^8^ including taurine and glycine conjugated bile acids (TCBA, 3.08 ppm, and GCBA, 3.74 ppm, respectively). More recent MRS studies of healthy subjects and patients with PSC were performed at 3T.^9^, ^10^

Ultrahigh field of 7T has promising advantages for MRS, such as high spectral resolution and higher signal‐to‐noise ratio (SNR), which could help better resolve bile components and acquire spectra faster.

Relaxation correction, with longitudinal (T_1_) and transversal (T_2_) relaxation times, is necessary for the calculation of accurate bile component quantification by MRS. T_2_ relaxation times of bile components were reported *in vitro* from porcine bile^9^, ^11^, ^12^ and from bile of dogs.^13^ There is a need for assessment of human bile relaxation times as well. Studies in humans employed point‐resolved spectroscopy sequence (PRESS)^14^ with echo times (TE) of 30 msec and 60 msec.^7^, ^9^ These TEs are suitable for measurements of longer relaxing bile components, such as CCPL; however, a sequence with a TE < 30 msec would be advantageous for assessment of the short relaxing components. A modified ultrashort‐TE stimulated echo acquisition mode (STEAM) sequence has been used for liver MRS at 7T.^15^

Since the T_2_ relaxation time of water in gallbladder is several fold longer than the T_2_ relaxation time of water in the liver,^9^, ^16^ analyzing the water linewidth in water unsuppressed spectra could provide unique and definitive information about the origin of the signal and guide signal discrimination during postprocessing.

The aims of this study were to establish a measurement and postprocessing protocol for *in vivo* measurement of concentrations of human bile components corrected for T_2_ relaxation times measured in healthy subjects with ultrashort TE MRS during free breathing without water suppression at 7T and to apply the protocol in patients with cholangitis.

## Materials and Methods

### *Human Subjects*

The study protocol was approved by the Institutional Review Board and the Ethics Committee, and written, informed consent was obtained from all participants prior to enrollment in the study. *In viv*o MRS measurements were performed on 10 healthy volunteers (age 33.5 ± 4.1 years, body mass index [BMI] 22.4 ± 1.3 kg.m^–2^, five males and five females), two patients with primary biliary cholangitis (PBC) (age 47 and 63 years, BMI 24.7 kg.m^–2^ and 31.2 kg.m^–2^, one male and one female), and one patient with primary sclerosing cholangitis (PSC) (age 47 years, BMI 22.0 kg.m^–2^, female).

The healthy subjects had no history of gallbladder disease. The subjects had no history of acute, chronic liver disease or inflammatory bowel disease. None of the subjects reported any special dietary restrictions.

### In Vivo *Gallbladder MRS*

All MRS experiments were performed with a 7T Magnetom MR system (Siemens Healthineers, Erlangen, Germany) with a double‐tuned ^1^H/^31^P surface coil (^1^H double loop 14 × 23 cm, Rapid Biomedical, Rimpar, Germany). All subjects were scanned in the supine position after overnight fasting. Fasting was confirmed by imaging a full gallbladder on the T_1_‐weighted scout images. The center of the coil was placed over the lowest ribs, on the right side of the rib cage.

The spectroscopic voxel was positioned according to T_1_‐weighted scout images (gradient echo, relaxation time / echo time [TR/TE] = 7.8/3.7 msec, field of view = 400 × 400 mm^2^, slice thickness = 6 mm). The frequency was adjusted on the water signal, and the homogeneity of the B_0_ field was first improved with an automatic B_0_ shimming method calculated from gradient echo images. Linear shims were then manually adjusted in an interactive mode. The accepted water linewidth was 60 Hz or less in magnitude mode.

For MRS, a STEAM sequence with hermite pulses (bandwidth of 3.57 kHz) and spoiler gradient switching^15^ was used. The signal was measured with mixing time (TM) of 10 msec and acquired with 1024 complex points, and the receiver bandwidth was set to 3000 Hz. TR was set to 5 seconds. No outer volume suppression was used. The reference voltage for RF pulses was adjusted according to the water signal amplitude by incrementally changing the voltage of the RF pulses from 280–340 V. More information about the sequence, RF pulses, and their adjustment can be found elsewhere.^15^ Due to the narrow frequency profile of hermite pulses and chemical shift displacement error (CSDE), an acquisition with a frequency offset set on water (0.0 ppm) was used for quantification of signals from 8 ppm to 3 ppm, and a separate acquisition with an offset of –3.4 ppm from water resonance was used for quantification of signals from 3 ppm to 0 ppm. The size and the position of the voxel were individually adjusted according to the size of the gallbladder, with the size ranging from 1.0–3.4 cm^3^ (mL). The linewidth of the water signal was observed on a real‐time display. The localization of a voxel was considered to be accurate when a series of relatively narrow water peaks was detected. The number of spectral transients per frequency offset (NT) was 8. Spectral transients were stored individually.

Every healthy subject was first measured with a TE of 6 msec, 50 msec, 100 msec, and 150 msec. Additional measurements with TE of 20 msec, 30 msec, 70 msec, 120 msec, and 200 msec were performed, according to subject compliance. Patients were measured only with a TE of 6 msec. The total acquisition time per one TE was 1.33 minutes. Total examination time including patient positioning, shimming, and sequence adjustments ranged from 15–20 minutes.

### *Data Postprocessing*

Signals were processed offline with in‐house software written in MATLAB (MathWorks, Natick, MA). A Fourier transform was applied to all eight individual spectral transients per TE and frequency offset, the full‐width at half‐maximum of water (FWHM_H2O_) was calculated from magnitude spectra, and spectra with FWHM_H2O_ <60 Hz (equivalent of 30 Hz in absorption mode) were selected for further signal processing. The FWHM_H2O_ limit of 60 Hz in magnitude mode was used for processing of all signals. The selected transients were automatically phased, frequency aligned, and averaged. The resulting spectrum was truncated (last 200 complex points) and zero‐filled to 4096 complex points in jMRUI.^17^

The chemical shifts of bile components were obtained from the literature^3^, ^18^ and used for peak identification: amide protons of glycine and taurine conjugated bile acids (NHCBA), 8.00 ppm; olefinic lipids and cholesterol (OLC), 5.35 ppm; methylene protons (ML), 1.26 ppm; methyl signals of bile acids, lipids and cholesterol (BALC1.0), 1.00 ppm; methyl signals of bile acids, lipids and cholesterol (BALC0.9), 0.88 ppm; and methyl signal of total bile acids and cholesterol (TBAC), 0.66 ppm.

The signals were fitted with Lorentzian line shapes using the AMARES algorithm.^19^ Because no water suppression was used during the data acquisition, the water signal was removed offline with the Hankel–Lanczos singular value decomposition (HL‐SVD) method^20^ in jMRUI. This method was successfully applied in the *in vivo* MRS study at 7T.^15^ In case the water sidebands were not efficiently removed, the HL‐SVD method was used to remove them at a chemical shift of ±553 Hz (±1.9 ppm) from the water peak.

### *T_2_ Relaxation Times*

Monoexponential T_2_ relaxation was assumed for all bile components. Individual T_2_ relaxation times were calculated in MATLAB with the following monoexponential fitting function:

(1) $$S\left(T_{E}\right)=S_{0}\exp\left(–T_{E}/T_{2}\right)$$

where S_0_ represents the signal intensity at TE = 0 msec. The coefficient of determination (R^2^) was used as an indicator of the quality of the fit.

Mean T_2_ relaxation times of bile components were calculated from the individual T_2_ relaxation times from all subjects. The calculation of T_2_ relaxation times are described in more detail in the Supplemental Material (S2). The T_2_ relaxation times are reported as mean ± standard deviation and range.

## Concentrations of Bile Components

Concentrations in mM were calculated according to Mohajeri et al and Fayad et al^9^, ^21^:

(2) $$\text{Concentration}=\frac{I_{\mathrm{BC},T2}}{I_{H2O,T2}}\frac{{\mathrm{NP}}_{H2O}}{{\mathrm{NP}}_{\mathrm{BC}}}\frac{10^{6}}{M_{H2O}}{\mathrm{CF}}_{H2O}$$

where I_BC,T2_ and I_H2O,T2_ are the corrected signal intensities for individual T_2_ relaxation times for bile components and water, respectively; NP_BC_ and NP_H2O_ are the number of protons for bile component and water (NP_H2O_ = 2), respectively; M_H2O_ is the molar mass of water (≈18.015 g/mol); and CF_H2O_ is a conversion factor for the water content of human gallbladder bile (CF_H2O_ = 0.9). The concentrations are reported as mean ± standard deviation and range.

## Results

### In Vivo *MRS*

**Figure 1:**
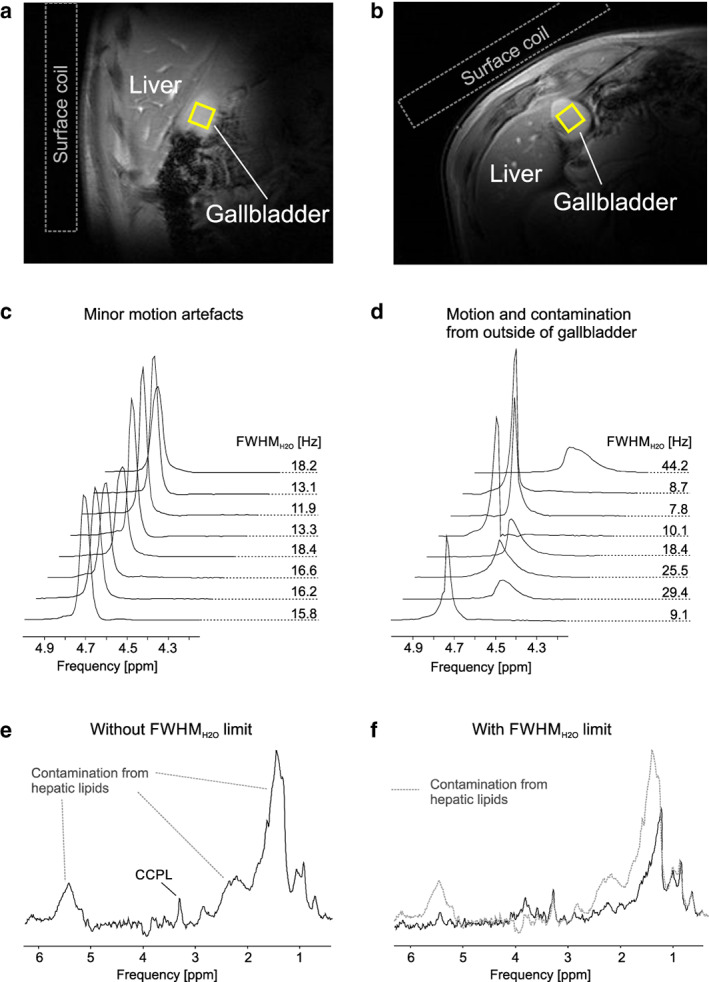
Example of voxel placement and signal acquisition. Localizer images with 1 mL voxel placed inside of the gallbladder (solid yellow lines) with position of the surface coil (represented by the dashed lines) in the coronal (**a**) and axial planes (**b**). Effects of minor motion artifacts on the water linewidth in the gallbladder (**c**). Major motion artifacts contain visible differences in water peak shape, amplitude, and chemical shift (**d**). Postprocessing of the spectra shown in (d) without application of FWHM_H2O_ result in signal contamination from hepatic water and lipids (**e**). Postprocessing of the spectra shown in (d) with application of FWHM_H2O_ limit result in better resolution of bile components (**f**). Please note that the measurement shown in (d) was repeated with voxel repositioning within the same subject and the data were used for final analysis. All examples of spectra from gallbladder were measured with a TE of 6 msec and with frequency offset of –3.4 ppm. (Spectra without water signal (e,f) were apodized with a 5 Hz Lorentzian filter for illustrative purposes.)

An example of surface coil position and placement of voxels and gallbladder MRS are shown in Fig. 1a,b. Mean size of the voxel in healthy subjects was 2.9 ± 1.0 mL. This limit allowed keeping on average at least 50% of the transients with average FWHM_H2O_ from 15.0 Hz to 20.2 Hz. An overview of the number of subjects measured per specific TE, mean number of accepted scans per frequency offset and TE, and FWHM_H2O_ per frequency offsets and TE, is summarized in Tables S1 and S2 in the Supplemental Material.

**Figure 2:**
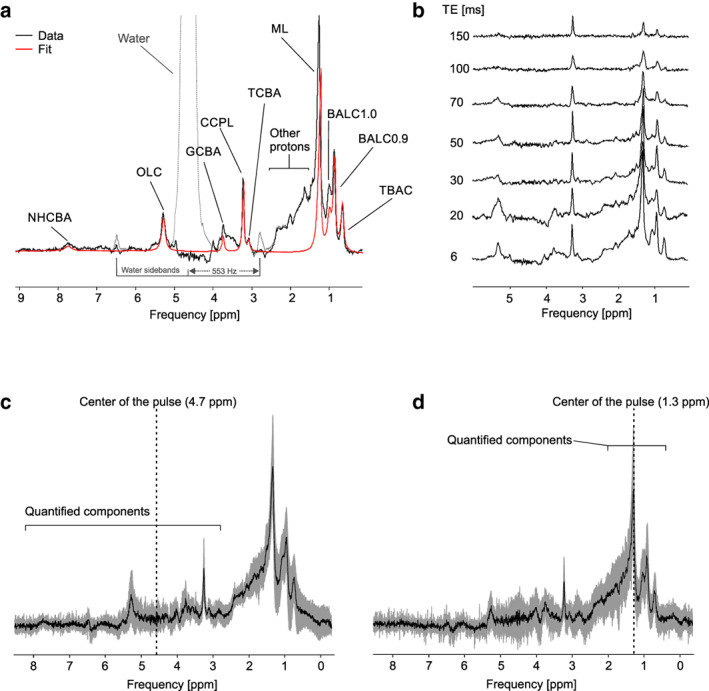
Examples of postprocessed bile spectra from healthy volunteers. Spectrum with removed water signal and water sidebands (NT = 8, TE = 6 msec, frequency offset of 0.0 ppm) with spectral fit (red) (**a**). The dashed lines in (a) represent spectrum with unremoved water sideband peaks at 2.8 ppm and 6.6 ppm after deficient spoiler gradient cycling. Example of gallbladder spectra measured with different TEs for calculation of T_2_ relaxation times (**b**). Standard deviations (shaded area) with the average spectrum of all 10 healthy subjects from the gallbladder obtained with the STEAM sequence (TE = 6 msec) with frequency offsets of 0.0 ppm (**c**) and –3.4 ppm (**d**)

An example of the bile spectrum measured at 7T is depicted in Fig. 2a. An example of spectra used for the calculation of T_2_ relaxation times for bile components is depicted in Fig. 2b. Mean spectra of all subjects measured with TE of 6 msec are depicted with their respective standard deviations in Fig. 2c,d.

### *T_2_ Relaxation Times*

T_2_ relaxation times were calculated for all 10 bile components in all subjects except for NHCBA and OLC in one subject, due to low SNR. The T_2_ relaxation times of all bile components and water signals with corresponding R^2^ values are summarized in Table 1. Comparisons of previously published T_2_ relaxation times measured *in vitro* to T_2_ relaxation times measured in this study are shown in Table 2.

**Table 1:** Mean Values and Range of T_2_ Relaxation Times (msec) of 10 Bile Components Measured in Healthy Volunteers With Coefficients of Determination (R^2^)

Component	Chemical shift	Mean T_2_ (msec)	Range T_2_ (msec)	R^2^
NHCBA^a^	8.00 ppm	50 ± 29	21–108	0.704 ± 0.255
OLC^a^	5.35 ppm	55 ± 28	19–101	0.821 ± 0.118
Water	4.70 ppm	145 ± 38	104–241	0.942 ± 0.044
GCBA	3.74 ppm	68 ± 42	27–163	0.752 ± 0.179
CCPL	3.22 ppm	165 ± 59	100–268	0.729 ± 0.182
TCBA	3.08 ppm	122 ± 52	67–244	0.617 ± 0.201
ML	1.26 ppm	60 ± 14	42–85	0.923 ± 0.088
BALC1.0	1.00 ppm	40 ± 15	27–71	0.910 ± 0.096
BALC0.9	0.88 ppm	58 ± 12	42–73	0.886 ± 0.136
TBAC	0.66 ppm	33 ± 12	17–62	0.912 ± 0.099

Data are shown as mean with standard deviation.

^a^ This metabolite was measured in nine out of 10 healthy volunteers.

**Table 2:** Comparison of T_2_ Relaxation Times (msec) of Previously Measured Bile Components *In Vitro* in Animals for Different Magnetic Field Strengths (B_0_) With T_2_ Relaxation Times Measured in Humans *In Vivo* in This Study at 7T

	T_2_ (msec)			
B_0_	1.5T	2.35T	3.0T	7.0T
NHCBA			36	50
Water	372	153	172	145
GCBA			84	68
CCPL	178		155	165
TCBA			93	122
TBAC			25	33

The T_2_ of water and CCPL was measured at 1.5T in porcine bile^7^; the T_2_ of water was measured at 2.35T in canine bile^13^; and the T_2_ of water and the other five bile components was measured at 3.0T in porcine bile.^9^, ^11^, ^12^

### *Concentration of Bile Components*

The lowest mean concentration in healthy subjects was found in the case of CCPL, 42 ± 17 mM (range: 19–66 mM). The concentration of CCPL had the smallest standard deviation of all bile components. Other relatively low concentrated metabolites were TCBA and GCBA. Comparable concentrations were found in the case of TBAC, OLC, and BALC1.0. The highest concentrations were in the case of BALC0.9 and ML.

All patients had lower mean concentrations of bile components compared to healthy subjects, whereas the PSC patient had lower mean concentrations of bile components compared to mean concentrations of PBC patients. The mean concentrations of bile components with respective standard deviations and ranges measured in healthy subjects and patients are summarized in Table 3. Comparison of previously published concentrations of previously measured bile components in healthy subjects and in patients with subjects in this study is shown in Table 4.

**Table 3:** Mean and Range of Concentrations of Nine Bile Components (mM) Measured in 10 Healthy Volunteers, Two Patients With Primary Biliary Cholangitis (PBC), and One Patient With Primary Sclerosing Cholangitis (PSC)

Component	Mean healthy (*n* = 10)	Range healthy	Mean PBC (*n* = 2)	Range PBC	PSC (*n* = 1)
NHCBA^a^	93 ± 66	42–228	44 ± 15	33–55	9
OLC^a^	154 ± 124	30–369	110 ± 25	93–128	40
GCBA	67 ± 32	32–128	58 ± 33	35–82	54
CCPL	42 ± 17	19–66	26 ± 15	16–36	9
TCBA	48 ± 35	16–128	20 ± 20	6–33	18
ML	740 ± 391	148–1351	531 ± 298	320–742	265
BALC1.0	175 ± 92	30–352	72 ± 82	13–130	26
BALC0.9	260 ± 138	57–508	157 ± 31	135–179	58
TBAC	153 ± 90	36–320	50 ± 13	41–59	37

^a^ This metabolite was measured in nine out of 10 healthy volunteers.

**Table 4:** Comparison of Mean Concentrations (mM) of Previously Measured Bile Components in Healthy Subjects and in Patients With Subjects in This Study

	Healthy subjects			Patients with PSC	
	Prescot et al^7^	Mohajeri et al^9^	This study	Mohajeri et al^10^	This study
NHCBA		72	93	35	9
GCBA		55	67	20	54
CCPL	36	48	42	20	9
TCBA		26	48	11	18
TBAC		123	153	61	37

## Discussion

In the work presented here, the concentration and T_2_ relaxation rates of bile components were measured with *in vivo* MRS at 7T using a modified STEAM sequence with free breathing and an ultrashort TE without water suppression in 10 healthy subjects and three patients with cholangitis. The high spectral resolution of 7T enabled detection and quantification of water and other nine bile components. The detection of GCBA, TCBA, and methyl signals of bile acids, lipids, and cholesterol was superior to 3T.

Although prone positioning for gallbladder MRS has been proposed to minimize respiratory movements and susceptibility differences at tissue–air interfaces,^22^ the supine position used in our study provided data with sufficient quality. The mean size of the spectroscopic voxel in our study was similar to the mean voxel size reported by Prescot et al (3.3 mL).^7^

Acquisitions with breath‐hold and respiratory gating techniques are feasible and widely used for liver and gallbladder MRS,^9^, ^23^ yet there are several factors to consider. The placement of the volume of interest (VOI) depends on anatomical images acquired during the same respiratory phase. Even then, there is no guarantee that the prescribed VOI will be in the same location, since the subject's movement during the breathing phase could change the position of the VOI relative to the investigated tissue. This problem is more pronounced in the gallbladder than in the liver due to the nature of its small size and range of shapes.^24^ Previous approaches with MRS employed triggering techniques,^8^, ^9^ which enabled acquisition of the gallbladder spectra without contamination and high SNR. The triggering techniques use an effective TR that is adjusted to the breathing cycle of a subject. Our approach fixed the TR to 5 seconds, the average time for a breathing cycle.^9^ The choice of using a TR of 5 seconds was to minimize any possible T_1_ effects as well. According to measurements performed on porcine bile at 3T,^9^, ^11^, ^12^ the T_1_ relaxation time of water was 780 msec and the longest T_1_ relaxation time of other bile components was below 400 msec.

Abdominal MRS employing surface coils at 7T is particularly challenging due to the positioning, B_1_ power constraints and inhomogeneity,^25^ difference in relaxation times,^26^, ^27^ and respiratory motions. The modified STEAM sequence for 7T has been shown to be a reliable method for the measurement of hepatic lipids.^15^ We applied the same method for the gallbladder. Narrow spectral bandwidths and frequency ranges of RF pulses in this sequence needed to be compensated by two measurements with different frequency offsets.^15^ Although these two measurements doubled the acquisition time, this approach also minimized the CSDE, which can be substantial at 7T.^15^ For example, in the case of the 3.4 mL cubical voxel (1.5 × 1.5 × 1.5 cm^3^) and the RF pulse centered at the water frequency, the CSDE for CCPL was only 12.3%, but for NHCBA it was 27.4%. In the case the pulse was centered at ML, the CSDE for TBAC was only 4.9%. The weakness of quantification of the NHCBA signal is discussed below.

Due to short TEs and strong spoiler gradients used in this sequence, unsuppressed strong water signals cause the occurrence of water sidebands. The sidebands result from vibrations of the gradients^28^ and/or oscillation of the magnetic field.^29^ In an ideal case, the spoiler orientation switching method^30^ eliminates the sidebands, but only when pairs of transients with opposite spoiler gradient orientations are acquired. For example, a water sideband at 2.80 ppm may appear close to the TCBA peak (3.08 ppm) in the gallbladder spectra. The HL‐SVD method proved to be sufficient to remove the water sidebands in the case of the matching pairs of transients were not available.

Postprocessing of the data required an efficient FWHM limit for the water signal. Due to the significant differences in T_2_ relaxations of water in the liver^15^ and the gallbladder (12 msec vs. 145 msec, respectively), the water linewidth in the gallbladder spectra was considerably narrower. Although we did not measure B_0_ distribution in the abdomen, this difference seemed to be more significant than differences in B_0_ homogeneity. An MR cholangiopancreatography (MRCP) is based on heavily T_2_‐weighted pulse sequences, taking advantage of the inherent differences in T_2_ relaxations between stationary fluid‐filled structures in the abdomen (long T_2_ relaxation time) and neighboring soft tissue (short T_2_ relaxation time).^31^ The same principle was applied in our proposed MRS acquisition. The FWHM_H2O_ limit used in this study of 30 Hz showed to be sufficient for acquisition of data from gallbladder. Importantly, it worked for both acquisitions with different frequency offsets, where the number of accepted transients and FWHM_H2O_ were comparable. Variations of water amplitude were observed across all subjects. These individual differences in the water amplitude can be attributed to different positions of the gallbladder relative to the surface coil; various shapes of the gallbladder; the voxel size and its placement. We used an NT of 8 per offset, which effectively gives an NT of 16 for acquisition of all signals. To minimize these variations and get higher SNR, we recommend increasing the NT to 32. This allows for better selection of the spectral transients based on their FWHM_H2O_. The acquisition time in this study was shorter than in previous studies: 3.2 minutes at 1.5T^7^ and 22.89 minutes at 3T.^9^ The preparation time included in the total examination time can be beneficial for other measurements; for instance, abdominal MRI and ^1^H MRS of the liver. These measurements can be performed in the same session with the same setup, except for voxel adjustments in the case of MRS.

T_2_ relaxation times of bile components were previously measured *in vitro* only in animals.^9^, ^11^, ^13^, ^32^ We did not observe a large decrease of the T_2_ relaxation time of water at 7T compared to literature values,^9^, ^11^, ^13^, ^32^ which suggests mostly dipolar interactions of water molecules without a large contribution of diffusion and susceptibility effects.^27^ The exception is the T_2_ relaxation time of water in porcine bile measured at 1.5T published by Prescot et al.^7^ We did not include a possibility for J‐modulation of bile components in the calculations of T_2_ relaxation times for two reasons. First, because spin–spin J‐coupling interactions in bile components measured with STEAM have not yet been reported, so we decided to use the simpler approach for their assessment. Second, the high precision for assessment of T_2_ relaxations times was of less importance for quantification because we used very short TE. The mean coefficients of determination of all components were higher than 0.7, which suggests acceptable assessment of T_2_ relaxation with monoexponential decay. However, additional studies are needed to establish a standard range of T_2_ relaxation times of bile components in human gallbladder *in vivo* in both healthy subjects and in patients.

The concentration of CCPL is in agreement with previous *in vivo* studies in humans.^7^, ^9^ Although we reported the concentration of NHCBA, it must be noted that the chemical shift difference from water of this signal is 3.3 ppm, which at 7T represents substantial chemical shift displacement error (CSDE). Moreover, the frequency profile of the RF pulse will underestimate this signal, as described previously.^15^ Despite this suboptimal acquisition, this signal could still be detected and quantified because of the small voxel size, which resulted in a VOI that was within the gallbladder even for bile components with substantial CSDE.

The concentrations of all bile components had large variations across all healthy volunteers. Variations have been observed *in vitro* in bile^33^ and *in vivo* MRS in monkeys^8^ and in humans.^9^ Khan et al^34^ implied that these variations may be due to variations in diet, nutrition, use of drugs, and even exposure to different environmental factors that affect bile secretion and gallbladder motility. A greater number of subjects with controlled dietary environments and restrictions are needed to better characterize the variability of bile components. Future studies will need to focus on the reproducibility of the results as well. Moreover, the composition of hepatic (bile duct) vs. gallbladder bile may be of primary interest for the pathogenesis and treatment of bile duct diseases.

Lower concentrations of bile components in patients with PSC have already been reported.^10^ The authors of that study suggested that the reduction in bile acid secretion could indicate accumulation of bile acids in hepatocytes. The results reported in our study support previous findings. Changes in concentrations of bile salts and phospholipids and their ratios can increase bile salt toxicity and, thereby, contribute to bile duct injury, as seen in a range of hereditary and acquired bile duct diseases.^35^ Importantly, the stimulation of phospholipid secretion may be a key therapeutic mechanism of several established and new drugs such ursodeoxycholic acid, obeticholic acid (as well as other farnesoid X receptor agonists), and fibrates for treatment of cholangiopathies such as PBC and PSC.^2^ Moreover, impaired phospholipid excretion has been linked to a broad spectrum of hereditary and acquired hepatobiliary disorders,^35^ including nonanastomotic structures after liver transplantation and low phospholipid‐associated cholelithiasis syndrome. Notably, stimulation of biliary phospholipid excretion may be an important therapeutic mechanism in new drugs used for the treatment of cholangiopathies.^2^

Detection and quantification of phosphatidylcholine in the gallbladder and liver can be alternatively performed using phosphorus MRS imaging (^31^P MRSI) at 7T.^36^, ^37^ Phosphatidylcholine protects bile ducts from the harmful effects of bile acids by the formation of mixed micelles.^38^ The combination of MRS with ^31^P MRSI at 7T could provide an even more detailed picture of the gallbladder.

### *Limitations*

Detecting abdominal motions are one of the main limitations of this study. Our setup did not contain any gating technique to reduce motion artifacts. Although our approach was able to filter out signals that were not gallbladder‐specific, a combination of techniques for motion detection could increase the number of accepted transients per unit time. Future studies should focus on incorporating these techniques.

B_1_ field of the surface coil is another limitation of this study. The variation of signal amplitude was largely dependent on the anatomy of the subject: distribution and thickness of the adipose tissue and size and location of the gallbladder. Although the surface coil used in this study had an exceptional penetration metric, the position of the gallbladder could still be on the edge of reasonable signal detection. A coil array design for body MRI^39^ could be a solution for imaging the abdominal cavity and measuring the gallbladder using MRS at UHF in the future.

In some cases, due to CSDE, the NHCBA signal could be detected from different tissues or not at all. The large variations of this signal can be explained by different anatomical variations of the gallbladder. This is a weakness of our protocol. In the case that NHCBA is of high interest, the pulse needs to be centered on the NHCBA signal (offset = +3.3 ppm).

The number of healthy subjects and patients was relatively small. This work provided a basis for future gallbladder MRS experiments and the relatively small sample sizes served as proof of principles and not as a reference for concentrations of bile components.

## Conclusion

*In vivo* ^1^H MR spectroscopy of the gallbladder during free breathing without water suppression is feasible at 7T. A short acquisition time and ultrashort TE enabled measurements of T_2_ relaxation times of 10 bile components, including water. The methods used in this study may allow for *in vivo* measurement of bile components; however, additional work needs to be done to establish standard ranges and the repeatability of the method. Importantly, this work provided a protocol for designing future MRS investigations of the bile system *in vivo*.

## Disclosures

M.T.: Speaker for BMS, Falk Foundation, Gilead, Intercept, and MSD; advisory boards for Albireo, BiomX, Boehringer Ingelheim, Falk Pharma, Genfit, Gilead, Intercept, MSD, Novartis, Phenex, and Regulus. He further received travel grants from Abbvie, Falk, Gilead, and Intercept and unrestricted research grants from Albireo, Cymabay, Falk, Gilead, Intercept, MSD, and Takeda. He is also coinventor of patents on the medical use of norUDCA (filed by the medical University of Graz as previous employer).

## Supporting information


**Appendix S1**: Supporting information
