# Supplementary material for: In Vivo 1H MR Spectroscopy of Biliary Components of Human Gallbladder at 7T
Source: J Magn Reson Imaging. 2020 Jun 5;53(1):98–107. doi: 10.1002/jmri.27207 (PMC7754442; doi:10.1002/jmri.27207)
Supplement: Supplementary file 1 — Appendix S1: Supporting information [file JMRI-53-98-s001.docx]

**In Vivo ^1^H MR Spectroscopy of Biliary Components of Human Gallbladder at 7 T**

**Supporting Information**

**S1: Processing of Spectral Transients**

Following tables summarize processing of spectral transients of healthy subjects (Table 1) and patients (Table 2). Mean number of transients per offset shows mean of all accepted transients from all subjects measured with the same T_E_ and frequency offset. Similarly, mean FWHM_H2O_ shows mean FWHM_H2O_ of all accepted transients from all subjects measured with the same T_E_ and frequency offset. Mean values are reported with standard deviation as the mean ± standard deviation.

**Table 1**: Number of healthy subjects measured at different T_E_s for T_2_ relaxation time assessment, mean number of accepted transients per T_E_ and frequency offset (out of 8 transients) and mean water linewidths of all scans per T_E_.

| T_E_ [ms] | No. Subjects | Mean No. Transients | | Mean FWHM_H2O_ | |
| --- | --- | --- | --- | --- | --- |
|  |  | Offset: 0 ppm | Offset: -3.4 ppm | Offset: 0 ppm | Offset: -3.4 ppm |
| 6 | 10 | 6.4 ± 1.8 | 5.2 ± 2.4 | 17.5 ± 7.2 | 15.0 ± 6.0 |
| 20 | 7 | 7.0 ± 1.2 | 4.3 ± 2.7 | 18.3 ± 6.1 | 16.0 ± 6.7 |
| 30 | 7 | 6.6 ± 1.9 | 6.5 ± 2.3 | 14.2 ± 6.2 | 16.1 ± 5.8 |
| 50 | 10 | 6.8 ± 1.8 | 5.6 ± 2.1 | 16.4 ± 5.9 | 18.4 ± 6.6 |
| 70 | 8 | 6.9 ± 1.0 | 5.9 ± 2.6 | 16.4 ± 6.6 | 16.8 ± 6.3 |
| 100 | 10 | 6.5 ± 1.5 | 6.1 ± 1.5 | 16.8 ± 6.1 | 18.6 ± 6.3 |
| 120 | 6 | 7.0 ± 0.6 | 6.0 ± 1.8 | 15.0 ± 6.5 | 15.9 ± 6.3 |
| 150 | 10 | 7.0 ± 2.2 | 6.6 ± 1.4 | 15.6 ± 6.1 | 18.0 ± 6.9 |
| 200 | 5 | 5.0 ± 2.1 | 5.8 ± 2.2 | 19.9 ± 4.8 | 20.2 ± 6.3 |

**Table 2**: Number of measured patients, mean number of accepted transients per T_E_ and frequency offset (out of 8 transients) and mean water linewidths of all scans per T_E_.

| Disease | No. Patients | Mean No. Transients | | Mean FWHM_H2O_ | |
| --- | --- | --- | --- | --- | --- |
|  |  | Offset: 0 ppm | Offset: -3.4 ppm | Offset: 0 ppm | Offset: -3.4 ppm |
| PBC | 2 | 8 ± 0 | 8 ± 0 | 12.5 ± 7.7 | 11.3 ± 2.6 |
| PSC | 1 | 8 | 8 | 5.1 ± 1.7 | 9.7 ± 3.4 |

The mean number of transients is higher in patients than the mean number of transients in healthy subjects. The opposite true for the mean FWHM_H2O_. This can be explained by a chance and possible longer T_2_ relaxation times of water. To gain a better understanding of this difference, larger number of patients needs to be investigated.

**S2: T_2_ Relaxation of Bile Components**

T_2_ relaxation times were assessed for all ten bile components in all ten healthy subjects. In an ideal case, T_2_ relaxation of NHCBA, OLC, Water, GCBA, CCPL and TCBA signals would be fitted from spectra measured with frequency offset of 0 ppm, and ML, BALC1.0, BALC0.9 and TBAC signals would be fitted from spectra measured with frequency offset of -3.4 ppm. However, when fit of a T_2_ decay of bile component was not acceptable (R^2^ < 0.4), we chose the data measured with frequency offset with higher R^2^. This means that some signals were not measured in the voxel due to CSDE. Since we used FWHM_H2O_ limit, which, similarly to magnetic resonance cholangiopancreatography (MRCP), was based on longer T_2_ relaxation of water in the gallbladder, we assumed that only signals from gallbladder could be measured. This was only done for calculations of T_2_ relaxation times, not for quantification purposes. Number of T_2_ relaxation measurements per frequency offset and per bile component is shown in Table 3.

As it was shown in Table 1, T_2_ relaxation was measured from T_E_ of 6, 50, 100 and 150 in all ten healthy subjects. Measurements with other T_E_s were added into the protocol according to a subject compliance. The number of compliant subjects differed per T_E_ which affected the calculations of T_2_ relaxation times. Mean number and standard deviations of T_E_s used for T_2_ relaxation time calculation per bile component is shown in Table 3.

If signal intensities were 80% higher or lower than expected signal intensity predicted by the monoexponential relaxation model, then these signals were treated as outliers and were excluded from calculations. Outliers were attributed to insufficient spectral quality or peak fitting. Percentage of outliers were defined as number of outliers to all number of all signal intensities per bile component, and they are shown in Table 3.

**Table 3**: Number of measurements of T_2_ relaxation times of bile components per frequency offset, and mean number of T_E_s per calculation of T_2_ relaxation times and percentage of outliers for a bile component.

| Component | No. Measurements | | Mean No. T_E_s | Outliers [%] |
| --- | --- | --- | --- | --- |
|  | Offset: 0 ppm | Offset: -3.4 ppm |  |  |
| NHCBA | 10 | 0 | 6.8 ± 1.0 | 2.9 |
| OLC | 10 | 0 | 6.6 ± 1.0 | 5.7 |
| Water | 10 | 0 | 7.0 ± 1.1 | 0.0 |
| GCBA | 10 | 0 | 6.7 ± 1.1 | 4.3 |
| CCPL | 8 | 2 | 6.8 ± 1.4 | 2.9 |
| TCBA | 10 | 0 | 6.9 ± 1.1 | 1.4 |
| ML | 1 | 9 | 7.0 ± 1.1 | 0.0 |
| BALC1.0 | 0 | 10 | 7.0 ± 1.1 | 0.0 |
| BALC0.9 | 2 | 8 | 7.0 ± 1.1 | 0.0 |
| TBAC | 1 | 9 | 7.0 ± 1.1 | 0.0 |

T_2_ relaxation behavior and mean T_2_ relaxation times with their standard deviations are shown in Figure 1. As it can be seen in Figure 1, the standard deviations in T_2_ relaxations are proportional to intensities of the bile components in the spectra. Water signal had the lowest deviations and NHCBA signal had one of the largest.


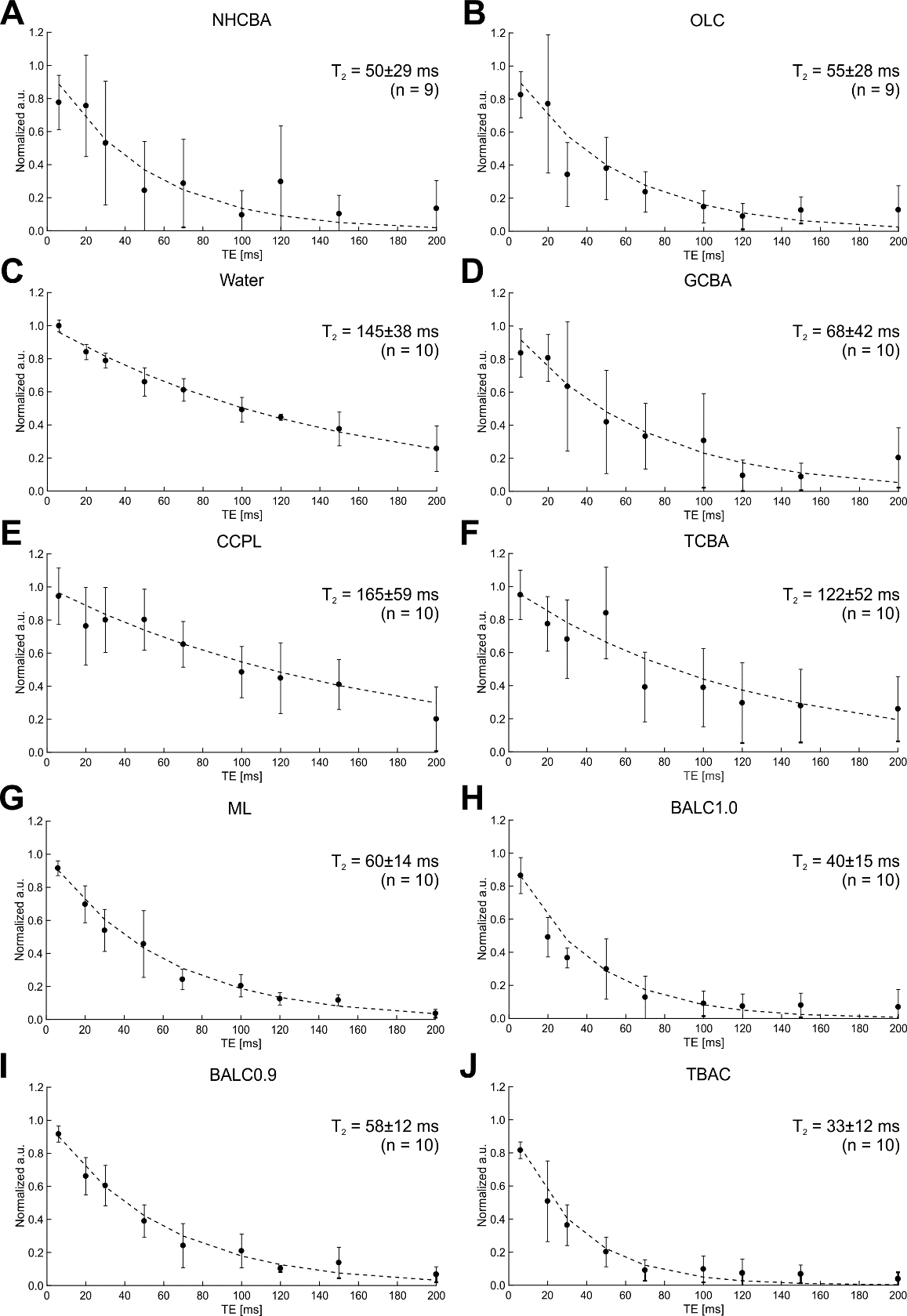


**Figure 1**: T_2_ relaxation behavior and the mean T_2_ relaxation times of ten bile components (A - J). For illustration purposes, all signals were normalized to the signal measured at T_E_ = 0 ms (M_0_ = 1). The dots represent average intensity per T_E_ for all subjects, the error bars represent standard deviation of the intensity per T_E_.
